# Chemical Composition of Essential Oil from *Apium graveolens* L. and Its Biological Activities Against *Sitophilus zeamais* Motschulsky (Coleoptera: Dryophthoridae)

**DOI:** 10.3390/plants14030347

**Published:** 2025-01-24

**Authors:** Ruchuon Wanna, Darika Bunphan, Benjapon Kunlanit, Phirayot Khaengkhan, Parinda Khaengkhan, Hakan Bozdoğan

**Affiliations:** 1 Department of Agricultural Technology, Faculty of Technology, Mahasarakham University, Kantarawichai District, Maha Sarakham 44150, Thailand; 2 Resource Management in Agricultural Technology Research Unit, Mahasarakham University, Kantarawichai District, Maha Sarakham 44150, Thailand; 3 Division of Plant Production Technology, Faculty of Agricultural Technology, Kalasin University, Kalasin 46000, Thailand; 4 Vocational School of Technical Sciences, Department of Plant and Animal Production, Kırşehir Ahi Evran University, Kırşehir 40100, Turkey

**Keywords:** Apiaceae, plant secondary metabolite, essential oil, stored insect pest, insecticide

## Abstract

The use of essential oils from certain herbal plants offers a promising alternative to synthetic insecticides for controlling the maize weevil, *Sitophilus zeamais* Motschulsky (Coleoptera: Dryophthoridae), a major pest that causes significant damage to stored grains. Essential oils, particularly from aromatic herbs in the Apiaceae family, are widely used in medicinal, cosmetic, and food industries and provided insecticidal properties to mitigate the environmental and health hazards associated with synthetic insecticides. This research aimed to investigate the insecticidal and repellent effects of *Apium graveolens* L. (celery) seed essential oil against *S. zeamais*. Chemical analysis of the commercially produced essential oil from *A. graveolens* seeds was conducted using a gas chromatograph–mass spectrometer (GC-MS), and the biological activity of the essential oil was determined by ingestion, contact, fumigation, and repellent tests. The analysis identified D-limonene (64.21%) and α-humulene (17.46%) as primary components of the oil. Toxicity assays revealed an observable contact toxicity, with higher concentrations and prolonged exposure increasing its effectiveness. The contact toxicity assays reported an LC_50_ of 19.83 nL/adult after 72 h. Additionally, the essential oil displayed repellent effects, effectively deterring weevils at concentrations above 16 µL/L air, but its feeding deterrence was weak. The essential oil’s strong insecticidal and repellent properties, which were concentration- and time-dependent, highlighted its potential as a sustainable alternative to synthetic pesticides for integrated pest management.

## 1. Introduction

*Sitophilus zeamais* Motschulsky (Coleoptera: Dryophthoridae) is a major pest of stored grains, including maize, rice, and wheat, causing significant crop losses due to both quantity and quality losses during storage. Both larvae and adults damage grains by direct feeding and contamination, resulting in weight loss, reduced germination, and reduced nutrient content [1]. Female *S. zeamais* lay eggs in grain bore holes to protect the developing larvae, making pest control a major challenge [2]. Infestations also promote an environment conducive to secondary pests, such as fungi and bacteria, further compromising the quality of stored products [3]. Losses due to *S. zeamais* are particularly severe during long-term storage, with post-harvest infestations reported to reduce grain weight by up to 59.48% within 90 days [4]. Since female *S. zeamais* lays eggs in grain bore holes, early detection is difficult, and pest management is complicated [5].

Traditionally, synthetic chemical insecticides, such as phosphine fumigants, have been the primary tool for controlling maize weevil infestations during storage [5,6]. Although effective, prolonged use of these chemicals has resulted in pest resistance and raised concerns about environmental and health hazards, such as chemical residues in food and soil and toxicity to non-target organisms [7,8,9]. Additionally, some chemicals, like methyl bromide, have been banned due to their environmental impact, prompting the search for safer alternatives [10]. As a result, there is growing interest in eco-friendly pest control solutions. Plant-based alternatives, particularly insecticidal essential oils, offer promising potential, providing lower toxicity, effective pest control, biodegradability, and minimal environmental impact [2,11].

Essential oils are natural plant extracts composed of volatile compounds and have gained attention as potential alternatives to synthetic insecticides. These oils are biodegradable and exhibit low toxicity to mammals, making them safer for both the environment and non-target species [2]. Essential oils contain complex mixtures of monoterpenes and sesquiterpenes, which function as toxins, insecticides, repellents, antifeedants, and oviposition inhibitors, disrupting pest behavior, development, and reproduction [12]. Notable examples include essential oils from clove, thyme, eucalyptus, and peppermint, which have demonstrated effectiveness against maize weevils through contact toxicity, fumigation, and behavioral disruption [9,13,14]. Despite their advantages, essential oils are volatile and prone to degradation, requiring optimal formulations to maintain sustained effectiveness [15].

*Apium graveolens* L. (celery) is an aromatic plant from the Apiaceae family, widely cultivated in temperate and subtropical regions worldwide, including Europe, Asia, Africa, and the Americas. It is a biennial or perennial herb with hollow stems, pinnate leaves, and small flowers arranged in umbels, and its seeds are rich in secondary metabolites [16,17]. Among these metabolites, D-limonene has gained attention for its neurotoxic effects on insects, disrupting olfaction and deterring feeding and oviposition [17,18]. Darmiati [19] reported that celery extracts inhibited egg-laying in beetles, highlighting their potential for integrated pest management (IPM). The accessibility and low toxicity of celery essential oil make it an attractive candidate for sustainable pest control. This study aimed to evaluate the insecticidal and repellent effects of *A. graveolens* essential oil on *S. zeamais* through contact, fumigation, and ingestion assays. The research also analyzed the chemical composition of the oil to assess its bioactivity and effectiveness. The objective was to determine the potential of *A. graveolens* essential oil as a sustainable alternative to synthetic insecticides, supporting its use in IPM strategies for the control of stored-product pests.

## 2. Results

### 2.1. Chemical Composition

The chemical analysis revealed 26 compounds, accounting for 97.23% of the overall composition of *A. graveolens* seed essential oil (Table 1). Six primary components (91.75%) had a percentage peak area greater than 1%, as identified by their highest peaks and classified using the International Union of Pure and Applied Chemistry (IUPAC) standard. D-limonene was the main component of *A. graveolens* seed essential oil, accounting for 64.21% of the total. The following other compounds were found in smaller amounts: α-humulene (17.46%), 3-butylisobenzofuran-1(3H)-one (3.02%), 4,11-selinadiene (2.44%), pentylbenzene (2.20%), and senkyunolide (2.42%). Monoterpene hydrocarbons represented 65.18% of the chemical composition, which consisted mainly of these compounds.

### 2.2. Ingestion Effect

The feeding deterrence potential of *A. graveolens* essential oil appeared limited. At a concentration of 9.375 µL/g flour, the feeding deterrence index (FDI) was 21.86%, reducing food intake to 20.90 ± 1.41 mg from 26.83 ± 1.52 mg in the control (0 µL/g flour). A higher concentration of 18.75 µL/g flour further reduced food consumption to 17.05 ± 2.27 mg, yielding an FDI of 28.30%. Both FDI values indicated weak feeding deterrence, as they fell within the 20–50% range (Table 2). Moreover, statistical analysis showed no significant difference in feeding deterrence, suggesting that reductions in consumption might have been due to random variation rather than a consistent inhibitory effect. The study suggested that *A. graveolens* seed oil may not be a potent feeding deterrent at the tested concentrations, though it demonstrated some potential for reducing feeding behavior.

### 2.3. Contact Effect

**Figure 1:**
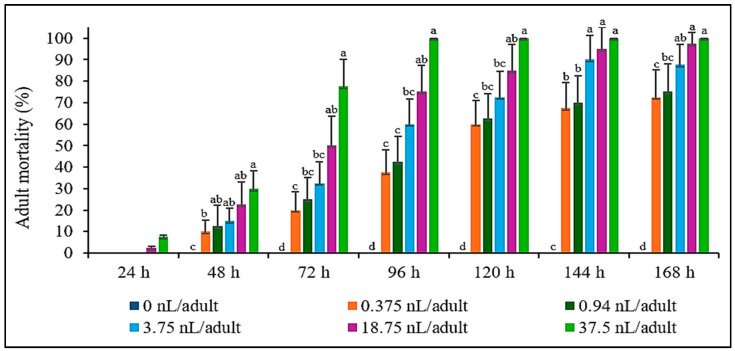
The adult mortality of *S. zeamais* after contact with essential oil of *A. graveolens* seeds. There were no significant differences observed in the mortality within 24 h (*p* > 0.05). The significant differences were found within 48–168 h (*p* < 0.01). Means of the same period followed by the same letter were not significantly different (LSD: *p* > 0.05).

The contact toxicity results of *A. graveolens* seed essential oil showed positive insecticidal potential. The study demonstrated that the contact toxicity of essential oil toward *S. zeamais* was both concentration- and time-dependent. At 48 h, the LC_50_ was 56.65 nL/adult (95% CL: 33.59–138.57 nL/adult), which decreased significantly to 19.83 nL/adult (95% CL: 6.42–38.68 nL/adult) after 72 h (Table 3). This reduction in LC_50_ highlighted that prolonged exposure increased the lethality of the essential oil, indicating that the contact toxicity became more effective over time. Higher concentrations of the essential oil resulted in quicker and more complete mortality, with 100% mortality achieved at 37.5 nL/adult by 96 h. In contrast, lower concentrations required longer exposure times to reach significant mortality, with 0.375 nL/mg insect achieving 72.5% mortality after 168 h (Figure 1).

### 2.4. Fumigation Effect

**Figure 2:**
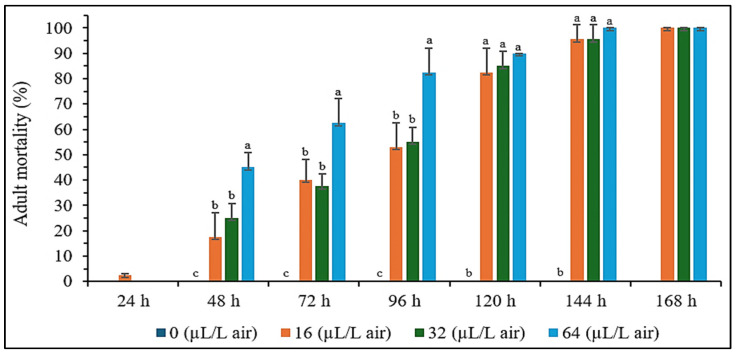
The adult mortality of *S. zeamais* after fumigation with essential oil of *A. graveolens* seeds. The data could not be statistically analyzed at 24 and 168 h. There were significant differences found within 48–144 h (*p* < 0.05). Means of the same period followed by the same letter were not significantly different (LSD: *p* > 0.05).

The fumigation effect of *A. graveolens* seed essential oil on *S. zeamais* was influenced by both concentration and exposure time. While the data at 24 and 168 h were not analyzed statistically, mortality increased significantly from 48 to 144 h. Higher concentrations, such as 64 µL/L air, caused faster mortality, reaching 100% by 144 h, while lower concentrations (16 µL/L air and 32 µL/L air) achieved complete mortality by 168 h (Figure 2). These results demonstrate the effectiveness of the essential oil as a fumigant, especially at higher doses, and its potential for controlling *S. zeamais* with extended exposure.

### 2.5. Repellent Effect

**Figure 3:**
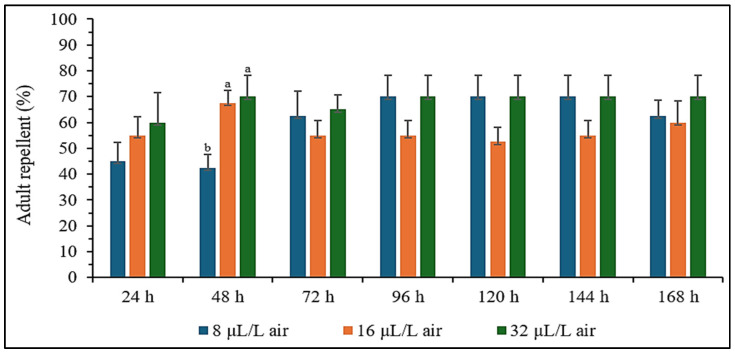
The adult of *S. zeamais* was repelled after fumigation with essential oil of *A. graveolens* seeds. There were no significant differences observed in the mortality within 24–168 h (*p* > 0.05), except significant differences were found within 48 h (*p* < 0.05). Means of the same period followed by the same letter were not significantly different (LSD: *p* > 0.05).

*Apium graveolens* essential oil acts primarily as a repellent to *S. zeamais*, with stronger effects at higher concentrations (16 and 32 µL/L air) across all time intervals. Initial attraction at lower concentrations (8 µL/L air) during the first 24 and 48 h shifted to repellency over time, suggesting that prolonged exposure enhanced the repellent properties of essential oil. Its sustained repellency after 72 h demonstrates the effectiveness of *A. graveolens* essential oil for long-term pest management, particularly at higher concentrations (Table 4). *Apium graveolens* essential oil effectively repels *S. zeamais* when used as a fumigant at 32 µL/L air, showing a consistent repellent effect over time. The absence of significant differences in repellency from 24 to 168 h suggests that the oil functions primarily as a repellent rather than a direct toxicant at this concentration. The significant difference observed at 48 h may indicate heightened sensitivity of the insects to the fumigant during this period (Figure 3). These findings confirmed that *A. graveolens* essential oil has potential as a long-term repellent for controlling *S. zeamais* populations, particularly by preventing infestation without causing immediate mortality.

## 3. Discussion

Generally, essential oils are highly complex mixtures of volatile compounds and may contain about 20–60 different components in varying concentrations, with some containing over 300 different compounds. Each essential oil is characterized by two or three of these components, which are usually present in a large proportion (20–70%), as compared to other components being present in small concentrations [21]. Typically, the major components of essential oils are the main components responsible for their biological properties. However, minor compounds may also play an important role in bioactivity, either by potentiating the action of major components or through antagonistic or additive effects [22]. This study showed that the main component of *A. graveolens* seed essential oil, according to the gas chromatograph–mass spectrometer (GC-MS) analysis, was D-limonene, which made up 64.21% of the total. Other compounds, present in smaller proportions, included pentylbenzene, senkyunolide, 4,11-selinadiene, 3-butylisobenzofuran-1(3H)-one, and α-humulene. This is consistent with other previously reported studies [23,24,25] that found primarily limonene concentrations of 58.38–76.9%. Because of its high limonene content, it has a strong and fresh scent note. This includes both the citrusy enantiomer (R)-(+)-limonene and the pine-fresh enantiomer (S)-(−)-limonene [26]. The essential oil from *A. graveolens* seeds is the most widely utilized in the pharmaceutical, flavor, fragrance, and aromatherapy industries [27,28,29]. However, this study showed notable deviations from earlier research. α-Humulene (17.46%) appeared prominently but was often absent or reported in lower amounts in other studies, where the chemical constituent was α-humulene (0.19%) in *A. graveolens* seed essential oil [25]. In addition, the presence of secondary compounds, such as 4,11-selinadiene and 3-butylisobenzofuran-1(3H)-one, detected in the essential oil of *A. graveolens* seeds in this study is noteworthy, as they have not been widely or consistently reported in previous studies [23,24,25]. The fragrance and chemical composition of essential oils can vary according to the geo-climatic location and growing conditions (soil type, climate, altitude, and amount of water available), season (before or after flowering), time of day when harvesting is achieved, genetic composition of the plant, plant part utilized (leaves, seeds, or stalks), type and method of preparation of the essential oil, etc. [30,31,32,33]. Therefore, all these factors influence the biochemical synthesis of essential oils in a given plant. Thus, the same species of plant can produce a similar essential oil, however, with different chemical compositions, resulting in different biological activities.

The essential oils of *A. graveolens* exhibited weak feeding deterrent effects, with FDI values ranging from 21.86% to 28.30%, suggesting that while the insects detected the oil, they did not entirely reject the food. These effects may be short-lived, as insects are highly adaptable and can initially be deterred but may recover their feeding behavior within hours, days, or even months [11]. Nevertheless, the feeding deterrent effects observed in this study could contribute to managing *S. zeamais*, a highly destructive pest known for its rapid grain consumption [34]. These findings align with those of Huang et al. [34], who reported that essential oil from *Myristica fragrans* Houtt. seeds reduced grain biomass and food consumption by *S. zeamais* adults in a flour disk bioassay. Similarly, de Lira et al. [35] demonstrated the ingestion toxicity of essential oils from the inflorescences of *Alpinia purpurata* (Vieill.) K. Schum. on *S. zeamais* adults, further supporting the potential of essential oils in pest control strategies. A limitation of this study is the absence of GC-MS analysis of the prepared substrate after incubation. Given the volatile nature of key compounds like limonene, changes in their proportions during preparation may have influenced the observed bioactivity. Without verifying the retention of these compounds, it is difficult to reliably attribute the effects to individual components. Future studies should include GC-MS analysis to confirm the chemical profile of the substrate post-incubation, ensuring a direct correlation between the observed effects and the presence of specific bioactive compounds. This approach will provide stronger evidence for the role of individual components in the essential oil’s efficacy and enhance its practical application in IPM strategies.

In the topical application assay, a known volume of an essential oil is applied directly to the insect’s body. The EO penetrates the cuticle to reach the haemolymph, which carries the EO to the target organs. The insect cuticle is made up of an outward hydrophobic thinner layer (epicuticle) and an internal hydrophilic thicker layer containing chitin (procuticle). The chitin fibers of the procuticle are predominant in hard-body insects (such as weevils), playing an important role in EO uptake; however, certain parts of the insect body, such as intersegmental membranes, sites around the setae, and sensilia, lack a developed chitin layer, offering less resistance to EO diffusion [36]. The essential oil of *A. graveolens* seeds demonstrated an LC_50_ of contact toxicity toward *S. zeamais* of 19.83 nL/adult in this investigation. The Apiaceae family, which includes spicy or aromatic plants with essential oils in various plant parts, includes *A. graveolens*. Additionally, some species in this family are known to be good sources of insecticidal essential oils [37,38]. Liu et al. [39] documented the toxicity of the Apiaceae plant *Ostericum sieboldii* essential oil. An LD_50_ value of 13.82 µg/adult indicated that the plant was extremely toxic to *S. zeamais* adults. Monoterpenes and sesquiterpenes are among the many compounds found in essential oils, which are composed of an abundance of secondary substances from aromatic families, like Lamiaceae and Apiaceae [11]. According to Tong and Coats [40], the insecticidal action of many plants that contain essential oil is caused by monoterpenoids, which also function as fumigants and contact toxicicants on many kinds of insect pests [41]. Contact toxicity effects on *S. zeamais* and other stored-grain pests have been reported for a variety of oils. The degree of toxicity has been attributed to traits like low-to-moderate volatility and ease of penetration of insect cuticle [42,43,44]. It is possible that the high concentrations and duration of *A. graveolens* essential oil allowed it to pass through the highly chitinized elytra of *S. zeamais*. However, the penetration capacity of the compound is not necessarily reflected in its bioactivity since many other chemical characteristics of the compounds influence their insecticidal properties [45].

Fumigation is the most widely used method of pest control in stored items because it is typically inexpensive, quick, efficient against insects at all life stages, and may be applied directly to insects [46,47]. Monoterpenoids, such as limonene, γ-terpinene, α-pinene, and thymol, have neurotoxic effects on various systems, including cholinergic, octopamine, and GABA-A receptors [40,47,48,49]. Essential oils containing monoterpenes, such as limonene, a key component of *A. graveolens* essential oil in this study, have been demonstrated to block octopamine receptors, leading to neurotoxicity, paralysis, and mortality in insects [50,51]. In this study, the higher concentrations (e.g., 64 µL/L air) caused 100% mortality within 144 h, while lower concentrations required 168 h to achieve the same effect. These findings were consistent with previous research highlighting the importance of concentration and exposure duration in effective fumigation toxicity. In addition, variations in chemical composition across studies may have influenced fumigant toxicity. For example, studies on *Foeniculum vulgare* and *Coriandrum sativum* revealed similar time- and dose-dependent effects but highlighted differences in toxicity levels, most likely due to variations in monoterpene and sesquiterpene content [52,53]. However, the essential oils obtained from many medicinal plants are rich in phytoconstituents, such as monoterpene hydrocarbons, oxygenated monoterpenoids, and sesquiterpene hydrocarbons. These compounds are known to have significant pharmacological and biological potential. Research has shown potential for antioxidant, antimicrobial, antiviral, insecticidal, and cytotoxic effects [54,55,56,57,58].

*Apium graveolens* essential oil is a potent long-term repellent against *S. zeamais*, particularly at higher concentrations (16–32 µL/L air). While lower concentrations initially attracted insects, prolonged exposure led to sustained repellency. Higher concentrations significantly enhanced the repellency rate, indicating a dose-dependent effect. *Apium graveolens* essential oil contains high levels of active compounds toxic to pest insects, functioning as both a contact pesticide and insect repellent [18,59,60]. The primary constituents include D-limonene, D-selinene, sedanolide, terpineol, santalol, selinene, nerolidol, pinene, and myrcene [61]. This study identified D-limonene as the major component (64.21%) of *A. graveolens* seed essential oil, consistent with prior findings that monoterpenes like limonene are effective insect repellents. D-limonene, acting as a repellent and insecticide, disrupts insects’ behavior by interfering with their olfactory systems rather than causing acute toxicity [62,63]. Tripathi et al. [64] reported that essential oils interact with larval olfactory receptors, blocking their sense of smell. The strong odor of *A. graveolens* has also been shown to repel larvae of *Spodoptera frugiperda*. The delayed repellency at lower concentrations in this study likely reflects concentration-dependent behavioral responses influenced by the oil’s composition and volatility. Variability in performance aligns with previous findings that chemical composition and environmental factors affect essential oil efficacy. Differences in compound concentrations, irrigation practices, and harvesting times impact the content and composition of celery essential oils. For instance, *A. graveolens* irrigated during harvest in September contained more limonene than unirrigated celery harvested in October in Poland [65].

This study found that *S. zeamais* exhibited concentration-dependent behavioral responses, which may explain the initial attraction at lower concentrations (8 µL/L air). Essential oils, particularly those high in volatile chemicals like D-limonene, can have varied effects depending on the concentration and exposure time. At low concentrations, the volatile components may initially stimulate the insects’ olfactory receptors, mimicking natural attractants. As these compounds accumulate and their volatility decreases, the repellent properties dominate, leading to a behavioral shift from attraction to repellency. This effect may also demonstrate how complex essential oils interact with insect odor systems. This is because different volatile parts of the oils have different effects on insects’ smell systems depending on their concentration gradients and how long they stay in the environment. Variability in environmental factors, such as humidity and temperature, might further influence the evaporation and perception of the essential oil, aligning with findings from previous studies [66].

The strong repellent properties of *A. graveolens* essential oil against *S. zeamais* demonstrated its potential as an eco-friendly tool in IPM for stored-grain protection. Its practical applications include use in fumigation systems, biodegradable sachets, or packaging materials to deter pests, while reducing reliance on synthetic pesticides. Nano/micro-formulation techniques, proven to enhance essential oil stability, volatility, and targeted delivery, represent a promising direction for future research to improve the essential oil’s practical efficacy. Integrating *A. graveolens* essential oil with advanced formulations and other IPM strategies can create a sustainable, comprehensive pest management solution for safer grain storage.

## 4. Materials and Methods

### 4.1. Insect Rearing

Adult *Sitophilus zeamais* Motschulsky were sourced from infested maize grains and reared in a plastic container (30 cm diameter and 50 cm height), as described by Wanna and Krasaetep [67]. They were sexed by examining the rostrum and abdominal shape of the insects under a stereomicroscope. The rostrum of the male is rough and distinctly shorter and wider than that of the female, while the rostrum of the female is smooth, shiny, and distinctly longer and narrower than that of the male [68]. A total of 150 pairs of *S. zeamais* were introduced into the container along with 1 kg of maize kernels for food. The container was sealed with a netted lid and maintained at a temperature of 30 ± 5 °C, with 70 ± 5% relative humidity and a 16 h light, 8 h dark photoperiod. This setup was contained in the laboratory of the Department of Agricultural Technology, Faculty of Technology, Mahasarakham University, Thailand. The insects were allowed to mate and lay eggs to increase their population for experimental consistency.

In this study, 7-day-old female *S. zeamais* were used, which were prepared by rearing their parents in plastic containers with sterile maize kernels as a food source. The sterile maize kernels were frozen for 12 h to eliminate any eggs, pests, or other contaminants [69]. Initially, the insects were allowed to reproduce for two generations to increase their population. In the third generation, 150 pairs of adult *S. zeamais* were released into the containers to mate and lay eggs for 7 days, after which the parent insects were removed. Only the maize kernels containing insect eggs were left in the containers. The containers were maintained at 30 ± 5 °C, with 70 ± 5% relative humidity and a 16 h light/8 h dark photoperiod. Newly emerged adults were observed daily and transferred to new containers with sterile maize kernels. The collection date of the insects was recorded. When the collected adults reached 6 days of age, they were identified and counted. If an insufficient number of female weevils were available for the experiment, these adults were used as new parents. If a sufficient number were available, the 7-day-old females were used in the bioassays.

### 4.2. Essential Oil Procurement and Chemical Composition Analysis

The essential oil extracted from *A. graveolens* seeds was obtained by steam distillation using a Clevenger apparatus for 3 h. During the distillation process, the plant material was placed on a sieve above a distillation vat. Water in the vat was heated, producing steam that carried the volatile compounds from the plant material. The steam and essential oil were condensed and separated to produce the essential oil. The collected essential oil was further separated from the aqueous phase, dried over anhydrous sodium sulfate, and stored in an amber bottle (Botanicessence Essential Oils, Bangkok, Thailand). It was kept at 4 °C in a dark environment until needed for bioassays.

The composition of the essential oil was verified through gas chromatography–mass spectrometry (GC-MS) analysis, following the methods outlined by Satongrod and Wanna [70]. The analysis was performed using a Clarus 680 gas chromatograph (PerkinElmer, Inc., Shelton, CT, USA) equipped with an Rtx-5MS capillary column (5% phenyl-methylpolysiloxane stationary phase, 30 m length, 0.32 mm diameter, and 1.0 µm film thickness) and a mass detector operating in electron impact (EI) mode. Helium served as the carrier gas at a flow rate of 1.0 mL/minute. Each sample (1 µL, 100,000 ppm) was injected in split mode with a split ratio of 1:100 (*v/v*). The injector temperature was maintained at 200 °C, and the oven temperature program began at 45 °C for 5 min, followed by a rise to 280 °C at 10 °C/minute, held for 5 min. The mass spectrometer operated at 70 eV in EI mode, using a quadrupole mass analyzer, and the detector temperature was set to 250 °C. Mass spectra were scanned (*m/z*) from 40 to 1000 amu.

The essential oil components were identified based on their retention indices, determined concerning the homologous series of C_7_–C_14_ (n-alkanes), by comparison of their mass spectra with the reports in the literature using the National Institute of Standards and Technology (NIST; Gathersburg, MD, USA) and Wiley version libraries (Scientific Instrument Services, Palmer, MA, USA) [71], ensuring a quality match of over 80%.

### 4.3. Ingestion Effect Bioassay

The ingestion effect of the essential oil was assessed using a modified version of the Xie et al. [72] method, as adapted by Napoleão et al. [73]. An artificial diet was created by suspending autoclaved wheat flour (2.0 g) in 5 mL of an essential oil solution (dissolved in acetone) to achieve final concentrations of 18.75 or 37.5 µL/g flour (µL of pure essential oil per gram of wheat flour). Five aliquots (200 µL) of the suspension were measured with a micropipette and applied to Petri dishes (90 mm diameter, with known weight) to form flour disks. These dishes were incubated overnight at 37 °C to dry. The dishes were weighed again to determine the final mass of the flour disks. Ten adult *S. zeamais* were introduced into each dish, with four replicates per bioassay. After 7 days at 30 ± 5 °C in complete darkness, the mortality rate and the weight of both the flour disks and insects were recorded. The control diet consisted of wheat flour suspended in acetone.

Using these results, the feeding deterrence and nutritional indices were calculated. The feeding deterrence index (FDI) was determined by the formula: FDI (%) = 100 × [(A − B)/A], where A represented the mass of food ingested by insects in the control, and B represented the mass of food ingested by insects in the test group [20]. Samples were categorized by FDI, as follows [74]: no feeding deterrence (FDI < 20%), weak (20% ≤ FDI < 50%), moderate (50% ≤ FDI < 70%), and strong (FDI ≥ 70%) feeding deterrence.

### 4.4. Contact Effect Bioassay

The contact effect assay followed the method outlined by Liu and Ho [75]. Essential oil from *A. graveolens* seeds was diluted in acetone to prepare test solutions at concentrations of 0 (control), 0.75, 1.88, 7.5, 37.5, and 75 µL/mL. Using a micropipette, 0.5 µL of each test solution was topically applied to the dorsal thorax of the insects. For the control group, acetone was used instead of the essential oil. Consequently, the test concentrations received by the insects were 0 (control), 0.375, 0.94, 3.75, 18.75, and 37.5 nL/adult, respectively. After treatment, the insects were placed in plastic containers (2.5 cm diameter and 5.5 cm length), and their mortality was monitored daily for seven days. Each experiment included four replicates.

### 4.5. Fumigant Effect Bioassay

The fumigation effect of *A. graveolens* essential oil was tested on adult *S. zeamais* using a modified method from Wanna and Krasaetep [67]. The experiment followed a completely randomized design (CRD) with four replicates for four essential oil concentrations: 0, 16, 32, and 64 µL/L air. The essential oil solutions were prepared by diluting them in 100% acetone. Strips of Whatman No. 1 filter paper (1.5 cm width and 5 cm length) were saturated with 200 µL of the essential oil solution, and the solvent was allowed to evaporate at room temperature for 2 min. These strips were then suspended in glass vials (2.5 cm diameter and 5 cm height) from the center of the screw caps of fumigation bottles (5.5 cm diameter and 10.5 cm height) to prevent contact with the insects. Ten adult *S. zeamais* were placed into each fumigation bottle, the caps were sealed, and the bottles were kept at 30 ± 5 °C with 70 ± 5% relative humidity and a 16 h light, 8 h dark photoperiod. For the control group, only acetone was used. The fumigation test lasted for 7 days, during which the bottles remained sealed to minimize the loss of volatile compounds from the essential oil. The bottles were not opened daily; instead, observations were conducted through the transparent walls of the containers to record insect mortality and behavior. The mortality of adult *S. zeamais* was recorded daily for 7 days. Insects were considered dead if no movement of their legs or antennae was observed.

### 4.6. Repellent Effect Bioassay

The repellent effect of *A. graveolens* essential oil against adult *S. zeamais* was evaluated using a vapor phase with choice test, based on a slightly modified version of the methods described by Satongrod and Wanna [70]. The test setup consisted of two plastic bottles (8 cm diameter and 17 cm height), designated as the test bottle and the control bottle. A small plastic tube (0.5 cm diameter and 15 cm length) connected the two bottles via a hole near the base of each. In the center of this tube, a hole was created for releasing adult *S. zeamais*, with a sliding tube mechanism to open and close the release point and prevent insect escape. The essential oil solutions were prepared at concentrations of 8, 16, and 32 µL/L air by diluting the oil in acetone. For each concentration, 100 µL of the solution was applied to a filter paper strip (1.5 cm width and 5 cm length) and allowed to evaporate for 2 min at room temperature. The treated filter paper was then placed inside a small glass vial (2.5 cm diameter and 5 cm height) and suspended from the center of the screw cap in the test bottle, which was then sealed tightly. In the control bottle, a filter paper strip was treated with 100 µL of pure acetone, following the same preparation steps. Ten adult *S. zeamais* were released into the connecting tube between the two bottles, and the sliding tube was securely closed. The number of weevils in both the test and control bottles was monitored daily for 7 days.

The repellency index (RI) was calculated following the method of Mazzonetto and Vendramim [76], using the formula: RI = 2T/(T + C), where T represented the percentage of *S. zeamais* in the bottle with essential oil, and C represented the percentage of *S. zeamais* in the control bottle. Standard deviations (SD) were also calculated, and the results were classified as follows: RI ± SD > 1 indicated an attractive effect, while RI ± SD < 1 indicated a repellent effect. Each test was performed with four replicates.

For the tests on the contact effect, fumigation effect, and repellent effect, *S. zeamais* were kept without food throughout the experiment. This design was chosen to isolate the effects of the essential oil and avoid any interference from food-related behaviors or interactions. While 7 days is a long duration, *S. zeamais* adults can survive for several days without food under experimental conditions. This approach is consistent with established protocols in similar studies to assess the direct effects of essential oils on insect mortality and behavior [75,77,78].

### 4.7. Statistical Analysis

The data were expressed as a mean of replicates ± standard errors. The mortality of adult *S. zeamais* was calculated using the formula: % adult mortality = (Nd/Nt) × 100, where Nd represented the number of dead adult *S. zeamais* and Nt denoted the total number of adult *S. zeamais* used in the test. Mortality data were adjusted for control mortality according to Abbott’s formula [79], where mortality in the control ranged from 5% to 20%. The contact toxicity of *A. graveolens* essential oil on adult *S. zeamais* was assessed for the concentration–mortality response using probit analysis with a reliability interval of 95% [80], yielding LC_50_ values and associated parameters. The repellent effect was assessed using the repellency index (RI) as the formula: RI = 2T/(T + C), where T represented the percentage of insects in the treatment bottle and C was the percentage of insects in the control bottle, with ranking values as follow [74]: RI ± SD > 1 was an attractive effect, while RI ± SD < 1 produced a repellent effect. Statistical data were obtained using the F-test by analysis of variance (ANOVA) based on the experimental plan factorial in CRD, and significant differences between treatment groups were analyzed using the least significant difference method (LSD < 0.05).

## 5. Conclusions

This study found that *A. graveolens* essential oil had effective insecticidal and repellent properties against *S. zeamais*. Key findings included notable fumigant and contact toxicity, with decreasing LC_50_ values over time, and concentration-dependent death. D-limonene and α-humulene are the major components responsible for its bioactivity. While the feeding deterrence was weak, the essential oil had sustained repellent effects, especially at higher concentrations, and demonstrated sustained repellency over lengthy periods. These findings highlight the oil’s potential as a long-term alternative to synthetic insecticides, particularly when used in IPM techniques for stored-goods pests.

## Figures and Tables

**Table 1:** The chemical composition of essential oil from *A. graveolens* seeds.

No.	Compound	Retention Index	RT	% Peak Area
1	α-Pinene	934	6.155	0.45
2	α-Myrcene	993	6.425	0.52
3	D-Limonene	1030	8.749	64.21
4	Carveol	1206	11.744	0.43
5	Pentylbenzene	1134	12.592	2.20
6	cis-p-Mentha-2,8-dien-1-ol	1165	13.366	0.08
7	cis-Dihydrocarvone	1180	14.139	0.13
8	trans-Carveol	1220	15.120	0.38
9	cis-Carveol	1225	15.658	0.17
10	Carvone	1242	16.078	0.27
11	Limonene-1,2-diol	1280	20.552	0.55
12	Valerophenone	1327	20.689	0.16
13	β-Elemene	1393	22.025	0.11
14	Caryophyllene	1418	23.249	0.48
15	α-Humulene	1456	26.732	17.46
16	4,11-Selinadiene	1495	26.877	2.44
17	2-Methyl-7-phenyl-2,6-heptanediol	1610	27.162	0.09
18	Alloaromadendrene	1455	27.317	0.14
19	2,2-Dimethylpropanoic acid, undec-2-enyl ester	1643	27.480	0.13
20	Cyclohexanebutanal, 2-methyl-3-oxo-, cis	1509	28.278	0.18
21	cis-p-Mentha-1(7),8-dien-2-ol	1206	29.146	0.31
22	Caryophyllene oxide	1582	29.739	0.70
23	3-Butylisobenzofuran-1(3H)-one	1658	32.734	3.02
24	Ledene oxide	1605	33.132	0.08
25	Senkyunolide	1558	35.134	2.42
26	2-Mehtyl-4-(2,6,6-trimethylcyclohex-1-enyl)but-2-en-1-ol	1641	39.912	0.12
	Chemical classes			
	Monoterpene hydrocarbons			65.18
	Oxygenated monoterpenes			2.01
	Sesquiterpene hydrocarbons			20.63
	Oxygenated sesquiterpenes			0.78
	Others			8.63
	Total			97.23

Retention index determined on an Rtx-5MS column. RT represents retention time.

**Table 2:** The feeding deterrence index (FDI) of adult *S. zeamais* after ingesting essential oil of *A. graveolens* seeds at seven days.

Concentration (µL/g Flour)	Mass of Food Ingested (mg)	%FDI	Reaction
0	26.83 ± 1.52	-	-
9.375	20.90 ± 1.41	21.86	weak
18.75	17.05 ± 2.27	28.30	weak
F-test	ns		

ns represents non-significant difference at *p* > 0.05. Feeding deterrence index (FDI) < 20% was no feeding deterrence, 50% > FDI ≥ 20% was weak, 70% > FDI ≥ 50% was moderate, and FDI ≥ 70% was strong feeding deterrence [20].

**Table 3:** Concentration–mortality response of adult *S. zeamais* after contact with essential oil of *A. graveolens* seeds.

Time (h)	n	LC_50_ (95% CL) (nL/adult)	Intercept	df	χ^2^
48	240	56.65 (33.59–138.57)	−1.333	19	213.95
72	240	19.83 (6.42–38.68)	−0.932	19	508.32

n = number of insects tested. CL = confidence limit. χ^2^ = chi-square.

**Table 4:** Repellency index (RI) of adult *S. zeamais* after fumigation with essential oil of *A. graveolens* seeds.

Time (h)	Concentration (µL/L Air)	Repellency Index (RI)	Reaction
24	8	1.1 ± 0.3	Attraction
	16	0.9 ± 0.3	Repellent
	32	0.8 ± 0.2	Repellent
48	8	1.2 ± 0.3	Attraction
	16	0.7 ± 0.3	Repellent
	32	0.6 ± 0.2	Repellent
72	8	0.8 ± 0.2	Repellent
	16	0.9 ± 0.1	Repellent
	32	0.7 ± 0.1	Repellent
96	8	0.6 ± 0.4	Repellent
	16	0.9 ± 0.1	Repellent
	32	0.6 ± 0.1	Repellent
120	8	0.6 ± 0.4	Repellent
	16	0.9 ± 0.1	Repellent
	32	0.6 ± 0.2	Repellent
144	8	0.6 ± 0.4	Repellent
	16	0.9 ± 0.1	Repellent
	32	0.6 ± 0.2	Repellent
168	8	0.8 ± 0.3	Repellent
	16	0.8 ± 0.2	Repellent
	32	0.6 ± 0.2	Repellent

Repellency index (RI) > 1 showed an attractive effect and RI < 1 showed a repellent effect.

## Data Availability

The original contributions presented in the study are included in the article. Further inquiries can be directed to the corresponding author.

## References

[1] Tefera T., Mugo S., Likhayo P., Beyene Y. (2011). Resistance of three-way cross experimental maize hybrids to post-harvest insect pests, the larger grain borer (*Prostephanus truncatus*) and maize weevil (*Sitophilus zeamais*). Int. J. Trop. Insect Sci..

[2] Patiño-Bayona W., Plazas E., Bustos J., Prieto J., Patiño-Ladino O. (2021). Essential oils of three *Hypericum* Species from Colombia: Chemical composition, insecticidal and repellent activity against *Sitophilus zeamais* Motsch. (Coleoptera: Curculionidae). Rec. Nat. Prod..

[3] Fleurat-Lessard F. (2016). Stored-Grain Pest Management. Encyclopedia of Food Grains.

[4] Costa S.J. (2014). Reducing Food Losses in Sub-Saharan Africa (Improving Post-Harvest Management and Storage Technologies of Smallholder Farmers).

[5] Herrera J.M., Zunino M.P., Dambolena J.S., Pizzolitto R.P., Gañan N.A., Lucini E.I., Zygadlo J.A. (2015). Terpene ketones as natural insecticides against *Sitophilus zeamais*. Ind. Crops Prod..

[6] Boyer S., Zhang H., Lempérière G. (2012). A review of control methods and resistance mechanisms in stored-product insects. Bull. Entomol. Res..

[7] Nayak M.K., Collins P.J., Throne J.E., Wang J.J. (2014). Biology and management of psocids infesting stored products. Annu. Rev. Entomol..

[8] Nwosu L.C. (2016). Chemical bases for maize grain resistance to infestation and damage by the maize weevil, *Sitophilus zeamais* Motschulsky. J. Stored Prod. Res..

[9] Yang Y., Isman M.B., Tak J. (2020). Insecticidal activity of 28 essential oils and a commercial product containing *Cinnamomum cassia* bark essential oil against *Sitophilus zeamais* Motschulsky. Insects.

[10] Arthur F.H. (2012). Aerosols and contact insecticides as alternatives to methyl bromide in flour mills, processing facilities, and food warehouses. J. Pest Sci..

[11] Isman M.B. (2006). Botanical insecticides, deterrents, and repellents in modern agriculture and an increasingly regulated world. Annu. Rev. Entomol..

[12] Mossa A.T.H. (2016). Green pesticides: Essential oils as biopesticides in insect-pest management. J. Environ. Sci. Technol..

[13] Boulogne I., Petit P., Ozier-lafontaine H., Loranger-merciris G., Boulogne I., Petit P., Ozier-lafontaine H., Desfontaines L., Loranger-Merciris G. (2012). Insecticidal and antifungal chemicals produced by plants: A Review. Environ. Chem. Lett..

[14] Singh K.D., Mobolade A.J., Bharali R., Sahoo D., Rajashekar Y. (2021). Main Plant volatiles as stored grain pest management approach: A Review. J. Agric. Food Res..

[15] Menossi M., Ollier R.P., Casalongué C.A., Alvarez V.A. (2021). Essential oil-loaded bio-nanomaterials for sustainable agricultural applications. J. Chem. Technol. Biotechnol..

[16] Nagella P., Ahmad A., Kim S.J., Chung I.M. (2012). Chemical composition, antioxidant activity and larvicidal effects of essential oil from leaves of *Apium graveolens*. Immunopharmacol. Immunotoxicol..

[17] Sellami I.H., Bettaieb I., Bourgou S., Dahmani R., Limam F., Marzouk B. (2012). Essential oil and aroma composition of leaves, stalks and roots of celery *(Apium graveolens* var. dulce) from Tunisia. J. Essent Oil Res..

[18] Tuetun B., Choochote W., Rattanachanpichai E., Chaithong U., Chaiwong P., Jitpakdi A., Tippawangkosol P., Riyong D., Pitasawat B. (2005). Repellent properties of celery, *Apium graveolens* L., compared with commercial repellents, against mosquitoes under laboratory and field conditions. Trop. Med. Int. Health.

[19] Darmiati N.N. (2013). Uji aktivitas ekstrak daun seledri (*Apium graveolens* L.) terhadap kumbang kacang *Callosobruchus chinensis* L. (Coleoptera: Bruchidae). Agrotop.

[20] Isman M.B., Koul O., Luczynski A., Kaminski J. (1990). Insecticidal and antifeedant bioactivities of neem oils and their relationship to azadirachtin content. J. Agric. Food Chem..

[21] Chouhan S., Sharma K., Guleria S. (2017). Antimicrobial activity of some essential oils-present status and future perspectives. Medicines.

[22] Bassolé I.H.N., Juliani H.R. (2012). Essential oils in combination and their antimicrobial properties. Molecules.

[23] Hassanen N.H.M., Eissa A.M.F., Hafez S.A.M., Mosa E.A.M. (2015). Antioxidant and antimicrobial activity of celery (*Apium graveolens*) and coriander (*Coriandrum sativum*) herb and seed essential oils. Int. J. Curr. Microbiol. App. Sci..

[24] Zorga J., Kunicka-Styczyńska A., Gruska R., Śmigielski K. (2020). Ultrasound-assisted hydrodistillation of essential oil from celery seeds (*Apium graveolens* L.). and its biological and aroma profiles. Molecules.

[25] Nouioura G., El fadili M., Ghneim H.K., Zbadi L., Maache S., Zouirech O., Danouche M., Aboul-Soud M.A.M., Giesy J.P., Lyoussi B. (2024). Exploring the essence of celery seeds (*Apium graveolens* L.): Innovations in microwave-assisted hydrodistillation for essential oil extraction using in vitro, in vivo and in silico studies. Arab. J. Chem..

[26] Parasuraman S. (2011). Prediction of activity spectra for substances. J. Pharmacol. Pharmacother..

[27] Khalil A., Nawaz H., Ghania J.B., Rehman R., Nadeem F. (2015). Value added products, chemical constituents and medicinal uses of celery (*Apium graveolens* L.)—A Review. Int. J. Chem. Biochem. Sci..

[28] Ambrose D.C., Manickavasagan A., Naik R. (2016). Leafy Medicinal Herbs: Botany, Chemistry, Postharvest Technology and Uses.

[29] Momin R.A., Ramsewak R.S., Nair M.G. (2000). Bioactive compounds and 1,3-Di[(cis)-9-octadecenoyl]-2-[(cis,cis)-9,12-octadecadienoyl] glycerol from *Apium graveolens* L. seeds. J. Agric. Food Chem..

[30] Sangwan N.S., Farooqi A.H.A., Shabih F., Sangwan R.S. (2001). Regulation of essential oil production in plants. Plant Growth Regul..

[31] Barra A. (2009). Factors affecting chemical variability of essential oils: A review of recent developments. Nat. Prod. Commun..

[32] Malhotra S.K. (2012). Celery. Handbook of Herbs and Spices.

[33] Manal A.S., Naglaa H.M.H., Mona H.M.A. (2015). Natural antioxidant changes in fresh and dried celery (*Apium graveolens*). Am. J. Energy Eng..

[34] Huang Y., Tan J.M.W.L., Kini R.M., Ho S.H. (1997). Toxic and antifeedant action of nutmeg oil against *Tribolium castaneum* (Herbst) and *Sitophilus zeamais* Motsch. J. Stored Prod. Res..

[35] de Lira C.S., Pontual E.V., de Albuquerque L.P., Paiva L.M., Paiva P.M.G., de Oliveira J.V., Napoleão T.H., do Amaral Ferraz Navarro D.M. (2015). Evaluation of the toxicity of essential oil from *Alpinia purpurata* inflorescences to *Sitophilus zeamais* (maize weevil). Crop Prot..

[36] Balabanidou V., Grigoraki L., Vontas J. (2018). Insect cuticle: A critical determinant of insecticide resistance. Curr. Opin. Insect Sci..

[37] Ebadollahi A. (2013). Plant essential oils from Apiaceae family as alternatives to conventional insecticides. Ecol. Balk..

[38] Zarshenas M.M., Samani S.M., Petramfar P., Moein M. (2014). Analysis of the essential oil components from different *Carum copticum* L. samples from Iran. Pharmacogn. Res..

[39] Liu Z.L., Chu S.S., Jiang G.H. (2011). Insecticidal activity and composition of essential oil of *Ostericum sieboldii* (Apiaceae) against *Sitophilus zeamais* and *Tribolium castaneum*. Rec. Nat. Prod..

[40] Tong F., Coats J.R. (2010). Effects of monoterpenoid insecticides on [3H]-TBOB binding in house fly GABA receptor and 36 cluptake in American cockroach ventral nerve cord. Pestic. Biochem. Phys..

[41] Rice P.J., Coats J.R. (1994). Insecticidal properties of several monoterpenoids to the house fly (Diptera: Muscidae), red flour beetle (Coleoptera: Tenebrionidae), and southern maize rootworm (Coleoptera: Chrysomelidae). J. Econ. Entomol..

[42] Sampson B.J., Tabanca N., Kirimer N., Demirci B., Baser K.H.C., Khan I.A., Spiers J.M., Wedge D.E. (2005). Insecticidal activity of 23 essential oils and their major compounds against adult *Lipaphis pseudobrassicae* (Davis) (Aphididae: Homoptera). Pest Manag. Sci..

[43] Ogendo J.O., Deng A.L., Kostyukovsky M., Ravid U., Matasyoh J.C., Omolo E.O., Kariuki S.T., Kamau A.W., Bett P.K., Shaaya E. (2011). Biocontrol potential of selected plant essential oil constituents as fumigants of insect pests attacking stored food commodities. Baraton Interdiscip. Res. J..

[44] Suthisut D., Fields P.G., Chandrapatya A. (2011). Fumigant toxicity of essential oils from three Thai plants (Zingiberaceae) and their major compounds against *Sitophilus zeamais*, *Tribolium castaneum* and two parasitoids. J. Stored Prod. Res..

[45] Dambolena J.S., Zunino M.P., Herrera J.M., Pizzolitto R.P., Areco V.A., Zygadlo J.A. (2016). Terpenes: Natural products for controlling insects of importance to human health—A structure-activity relationship study. Psyche.

[46] Graver J.E., Van S. (2004). Guide to Fumigation under Gas-Proof Sheets.

[47] Nenaah G.E. (2014). Chemical composition, toxicity and growth inhibitory activities of essential oils of three *Achillea* species and their nano-emulsions against *Tribolium castaneum* (Herbst). Ind. Crops Prod..

[48] Choi W.S., Park B.S., Lee Y.H., Yoon H.Y., Lee S.E. (2006). Fumigant toxicities of essential oils and monoterpenes against *Lycoriella mali* adults. Crop Prot..

[49] Tak J.H., Jovel E., Isman M.B. (2016). Comparative and synergistic activity of *Rosmarinus officinalis* L. essential oil constituents against the larvae and an ovarian cell line of the cabbage looper, *Trichoplusia ni* (Lepidoptera: Noctuidae). Pest Manag. Sci..

[50] Wang D.C., Qiu D.R., Shi L.N., Pan H.Y., Li Y.W., Sun J.Z., Xue Y.J., Wei D.S., Li X., Zhang Y.M. (2015). Identification of insecticidal constituents of the essential oils of *Dahlia pinnata* Cav. against *Sitophilus zeamais* and *Sitophilus oryzae*. Nat. Prod. Res..

[51] Qi X.J., Feng Y.X., Pang X., Du S.S. (2021). Insecticidal and repellent activities of essential oils from seed and root of celery (*Apium graveolens* L.) against three stored product insects. J. Essent. Oil Bear. Plants.

[52] Ebadollahi A. (2011). Susceptibility of two *Sitophilus* species (Coleoptera: Curculionidae) to essential oils from *Foeniculum vulgare* and *Satureja hortensis*. Ecol. Balk..

[53] Amini S.H., Tajabadi F., Khani M., Labbafi M.R., Tavakoli M. (2018). Identification of the seed essential oil composition of four Apiaceae species and comparison of their biological effects on *Sitophilus oryzae* L. and *Tribolium castaneum* (Herbst.). J. Med. Plants.

[54] Modaresi M., Ghalamkari G., Jalalizand A. (2012). The effect of celery (*Apium graveolens*) extract on the reproductive hormones in male mice. APCBEE Proc..

[55] Saini R.K., Song M.H., Yu J.-W., Shang X., Keum Y.S. (2021). Phytosterol profiling of Apiaceae family seeds spices using GC-MS. Foods.

[56] Kokotkiewicz A., Luczkiewicz M., Preedy V.R. (2016). Celery (*Apium graveolens* Vvar. *dulce* (Mill.) Pers.) oils. Essential Oils in Food Preservation, Flavor and Safety.

[57] Das S., Singh V.K., Dwivedy A.K., Chaudhari A.K., Upadhyay N., Singh A., Deepika, Dubey N.K. (2019). Antimicrobial activity, antiaflatoxigenic potential and in situ efficacy of novel formulation comprising of *Apium graveolens* essential oil and its major component. Pestic. Biochem. Physiol..

[58] Bat-Özmatara M. (2020). The antioxidant activity of Apium graveolens. Int. J. Food Eng..

[59] Kooti W., Daraei N. (2017). A review of the antioxidant activity of celery (*Apium graveolens* L). J. Evid. Based Complement. Altern. Med..

[60] Astuti R.D., Khotimah S. (2020). Patch formulation of celery leaves extract (*Apium graveolens* L.) as mosquito repellent. Adv. Soc. Sci. Educ. Humanit. Res..

[61] Al-Asmari A.K., Athar M.T., Kadasah S.G. (2017). An updated phytopharmacological review on medicinal plant of Arab region: *Apium graveolens* Linn. Pharmacogn. Rev..

[62] Luik A., Ochsner P., Jensen T.S. (1999). Olfactory responses of seed wasps *Megastigmus pinus* Parfitt and *Megastigmus rafni* Hoffmeyer (Hym., Torymidae) to host-tree odours and some monoterpenes. J. Appl. Entomol..

[63] Chaubey M.K. (2019). Essential oil as green pesticides of stored grain insects. Eur. J. Biol. Res..

[64] Tripathi A.K., Upadhyay S., Bhuiyan M., Bhattacharya P.R. (2009). A review on prospects of essential oils as biopesticide in insect-pest management. J. Pharmacogn. Phytother..

[65] Rozek E., Nurzyñska-Wierdak R., Salata A., Gumiela P. (2016). The chemical composition of the essential oil of leaf celery (*Apium graveolens* L. Var. Secalinum Alef.) under the plants’ irrigation and harvesting method. Acta Sci. Pol. Hortorum Cultus.

[66] Bedini S., Djebbi T., Ascrizzi R., Farina P., Pieracci Y., Echevrría M.C., Flamini G., Trusendi F., Ortega S., Chiliquinga A. (2024). Repellence and attractiveness: The hormetic effect of aromatic plant essential oils on insect behavior. Ind. Crops Prod..

[67] Wanna R., Krasaetep J. (2019). Chemical composition and insecticidal activity of essential oil from Indian borage against maize weevil. Int. J. Geomate.

[68] Ojo J.A., Omoloye A.A. (2012). Rearing the maize weevil, *Sitophilus zeamais*, on an artificial maize-cassava diet. J. Insect Sci..

[69] Wanna R., Khaengkhan P. (2023). Insecticidal activity of essential oil from seeds of *Foeniculum vulgare* (Apiales: Apiaceae) against *Sitophilus zeamais* (Coleoptera: Curculionidae) and its effects on crop seed germination. J. Entomol. Sci..

[70] Satongrod B., Wanna R. (2020). Chemical composition and bioactivity of essential oil from Indian borage (*Plectranthus amboinicus* (Lour.) Spreng) against *Callosobruchus maculatus* (F.). Int. J. Agric. Technol..

[71] Adams R.P. (2001). Identification of Essential Oil Components by Gas Chromatography/Mass Spectrometry.

[72] Xie Y.S., Bodnaryk R.P., Fields P.G. (1996). A rapid and simple flour-disk bioassay for testing substances active against stored-product insects. Can. Entomol..

[73] Napoleão T.H., Belmonte B.R., Pontual E.V., Albuquerque L.P., Sá R.A., Paiva L.M., Coelho L.C.B.B., Paiva P.M.G. (2013). Deleterious effects of *Myracrodruon urundeuva* leaf extract and lectin on the maize weevil, *Sitophilus zeamais* (Coleoptera, Curculionidae). J. Stored Prod. Res..

[74] Liu Z.L., Goh S.H., Ho S.H. (2007). Screening of Chinese medicinal herbs for bioactivity against *Sitophilus zeamais* Motschulsky and *Tribolium castaneum* (Herbst). J. Stored Prod. Res..

[75] Liu Z.L., Ho S.H. (1999). Bioactivity of the essential oil extracted from *Evodia rutaecarpa* Hook f. et Thomas against the grain storage insects, *Sitophilus zeamais* Motsch. and *Tribolium castaneum* (Herbst). J. Stored Prod. Res..

[76] Mazzonetto F., Vendramim J.D. (2003). Efeito de pós de origem vegetal sobre *Acanthoscelides obtectus* (Say) (Coleoptera: Bruchidae) em feijão armazenado. Neotrop. Entomol..

[77] Chu S.S., Liu Q.R., Liu Z.L. (2010). Insecticidal activity and chemical composition of the essential oil of *Artemisia vestita* from China against *Sitophilus zeamais*. Biochem. Syst. Ecol..

[78] González S., Pino O., Herrera R.S., Valenciaga N., Fortes D., Sánchez Y. (2011). Potencials of the powers of *Lonchocarpus punctatus* in the control of *Sitophilus zeamais*. Cuban J. Agric. Sci..

[79] Abbott W.S. (1925). A method for computing the effectiveness of an insecticide. J. Econ. Entomol..

[80] Finney D.J. (1971). Probit Analysis.

